# The Cost‐Effectiveness of a Multi‐Target Stool DNA‐Based Screening (COLOTECT), FIT, Colonoscopy and No Screening for Colorectal Cancer

**DOI:** 10.1002/cnr2.70176

**Published:** 2025-04-24

**Authors:** Junjie Huang, Mingtao Chen, Victor C. W. Chan, Xianjing Liu, Chaoying Zhong, Jianli Lin, Junjie Hang, Claire Chenwen Zhong, Jinqiu Yuan, Martin C. S. Wong

**Affiliations:** ^1^ Jockey Club School of Public Health and Primary Care, Faculty of Medicine The Chinese University of Hong Kong Hong Kong SAR; ^2^ Centre for Health Education and Health Promotion, Faculty of Medicine The Chinese University of Hong Kong Hong Kong SAR; ^3^ Department of Radiology and Nuclear Medicine Erasmus MC University Medical Center Rotterdam the Netherlands; ^4^ Department of Electrical Engineering and Automation Guangdong Ocean University Zhanjiang Guangdong China; ^5^ Peking‐Tsinghua Center for Life Sciences Academy for Advanced Interdisciplinary Studies, Peking University Beijing China; ^6^ Cancer Hospital & Shenzhen Hospital Beijing Chinese Academy of Medical Sciences and Peking Union Medical College China; ^7^ Clinical Research Center, Big Data Center The Seventh Afliated Hospital, Sun Yat‐Sen University Guangzhou Guangdong China; ^8^ The School of Public Health Peking University Beijing China; ^9^ The School of Public Health The Chinese Academy of Medical Sciences and The Peking Union Medical Colleges Beijing China; ^10^ The School of Public Health Fudan University Shanghai China

**Keywords:** colorectal cancer, cost‐effectiveness, multi‐target stool DNA, non‐invasive biomarker, screening

## Abstract

**Background:**

Around 1.9 million new cases and 1 million deaths worldwide were attributed to colorectal cancer (CRC) in 2020.

**Aims:**

The aims of this study are to assess the cost‐effectiveness of a multi‐target stool DNA‐based screening strategy, COLOTECT, compared to faecal immunochemical tests (FIT), colonoscopy, and no screening in the Asian population to inform more choices for policymakers in colorectal cancer screening.

**Method and Results:**

We assume that 100,000 persons aged 50 undergo annual FIT, annual COLOTECT multi‐target testing, or colonoscopies every 10 years until age 75. The data used in this study was retrieved from different sources including the Hong Kong Cancer Registry and previously published studies on the population aged 50 to 75 years old between 2010 and 2023. This study accessed the most cost‐effective screening strategy available. If a positive result of FIT or COLOTECT were observed, the participants would undergo a colonoscopy. The participants who used the colonoscopy as the main screening method conducted colonoscopies every 3 years. The Markov models were utilized to compare the outcomes from different strategies including life‐years saved, years of life lost, and incremental cost‐effectiveness ratio (primary outcome). The highest ICER was observed in colonoscopy (USD 160808), followed by FIT (USD 108952), and COLOTECT (USD 82206). A higher detection rate of CRC (COLOTECT: 39.3% vs. FIT: 4.5%), more CRC cases prevented (1272 vs. 146), and life‐years saved (2295 vs. 337) were observed in the COLOTECT strategy than in FIT. Additionally, a lower total cost per life‐year saved of COLOTECT (USD 180097) was observed than colonoscopy (USD 238356), which identified the more affordable and cost‐saving COLOTECT strategy.

**Conclusion:**

This study highlighted the better performance of COLOTECT than FIT in detecting CRC. Additionally, given its lower cost and higher acceptance, the COLOTECT strategy might be more cost‐effective than colonoscopy for massive CRC screening.

## 1 Introduction

Colorectal cancer (CRC) was the third most common cancer globally, with approximately 1.9 million new cases and 1 million deaths reported in 2020 [1]. In Hong Kong, CRC accounted for 41,450 (5.6 persons per 1000) prevalence cases, comprising 17.1% of total prevalence cases of all types of cancer as of 2022. In addition, it reported approximately 14.7% of new cancer cases and 15.4% of cancer‐related deaths, representing the second‐highest incidence and mortality rate among all cancer types in 2022 [2]. Numerous studies have demonstrated that screening with faecal immunochemical tests (FIT) and colonoscopy can significantly reduce CRC‐related mortality, which is 33% and 68%, respectively [3, 4, 5]. Although colonoscopy is considered the most sensitive screening method for CRC, it may be difficult to use in the front line due to its high cost, resource constraints, and low acceptance caused by patients' fear and aversion to invasive screening methods [6]. Studies based on the Hong Kong population indicate that lack of effective health insurance coverage and fear of colonoscopy hindered participants' acceptance of colonoscopy [7, 8]. Previous research has highlighted that the participation rates in FIT‐based screening programs are relatively low, with 54% globally. This can be attributed to the inability of FIT to detect adenomatous polyps, which is poor or limited sensitivity for serrated class precursor lesions, and its relatively low sensitivity for CRC (0.79, 95% CI 0.69 to 0.86) [9, 10, 11, 12]. Further investigation and preventive measures based on the Asian population are necessary due to an increasing trend of CRC incidences observed [13, 14].

For individuals who prioritize early detection of colorectal adenomas, a sensitive non‐invasive biomarker testing offers an appealing alternative to early detection and endoscopic polypectomy to reduce the risk of CRC progression [15, 16]. Screening by a non‐invasive method may serve as a useful method to enhance participation due to the higher acceptability [17]. COLOTECT is a novel, multi‐target stool DNA‐based non‐invasive test for CRC screening that detects abnormal genes by analyzing Syndecan‐2 (SDC2), Alcohol dehydrogenase iron‐containing 1 (ADHFE1) and Protein phosphatase 2A regulatory subunit B'gamma (PPP2R5C) in DNA methylation. The sensitivity and specificity of CRC were 88.0% and 92.0%, respectively [18], which is higher sensitivity (73.3%) and lower specificity (96.4%) than FIT [12]. Previous cost‐effectiveness analyses (CEA) studies have indeed predominantly focused on invasive screening methods like colonoscopy [19, 20, 21, 22, 23, 24, 25, 26, 27]. Furthermore, few studies have investigated the cost‐effectiveness of FIT [28, 29, 30]. However, there is a notable gap in economic analyses concerning non‐invasive biomarkers for CRC screening, especially for the novel technique, COLOTECT, which may hinder the implementation of population‐based CRC screening programs utilizing non‐invasive biomarkers. The objective of this study is to compare the cost‐effectiveness of FIT, COLOTECT, and colonoscopy for CRC screening, respectively, based on modeling data and assumptions. Ultimately, this study could serve as a reference for policymakers for better distribution of resources in providing better screening methods to improve patient care in Hong Kong.

## 2 Materials and Methods

### 2.1 Health Economic Analysis Plan

In this study, the Markov model was used as a decision analysis algorithm to analyze the data (see Figure S1). The Markov model adopted in this study followed a previous CEA conducted by Dr. J. J. Y. Sung and colleagues [31]. The sensitivity and specificity of the tests were incorporated into the model by using these parameters to determine the probabilities of true positive and true negative results for each screening strategy. Specifically, we used published data to define the sensitivity and specificity of COLOTECT, FIT, and colonoscopy. These parameters informed the transition probabilities within the Markov model, allowing us to accurately simulate the outcomes based on the likelihood of correct test results. The Microsoft Excel spreadsheet editor version 2108 was used to do the simulation.

### 2.2 Study Population

The study population consisted of 100,000 individuals who were 50 years old and had no previous history or symptoms of CRC. The cohort consists of 100,000 simulated individuals aged 50 to 75, representing average‐risk patients. This simulation is standard practice for cost‐effectiveness analysis (CEA). The population is evenly split, with 50% males and 50% females. Regarding the size of the population, we selected 100,000 patients to ensure sufficient statistical power for our analysis while maintaining practical feasibility for simulation. However, we did not perform any subgroup analyses in this study. The decision was based on the aim of maintaining a broad assessment of the overall study population without introducing additional complexity that could arise from subgroup comparisons. Three different screening strategies were evaluated: COLOTECT, FIT, and colonoscopy. The no screening strategy was used as a reference for comparing different strategies. Most countries did have CRC screening programs for patients equal to or over 50 years of age [32]. However, these programs normally target high‐risk populations and are a voluntary program. Therefore, this study used ‘no screening’ as the baseline. The screening strategies were applied to the entire hypothetical population and followed up until the age of 75 years.

#### 2.2.1 Setting and Location

The study population is located in Hong Kong. The healthcare is accessed by professional health care providers in public settings, hospital under the Hospital Authority. Treatments are funded by the government.

#### 2.2.2 Comparators

Currently, the Hong Kong government suggested that asymptomatic Hong Kong residents aged between 50 and 75 undergo Faecal Occult Blood Test (FOBT), a test that also detects blood in stool, and the substantial colonoscopy would be referred by Primary Care Doctors [33]. Hence, the primary comparator is ‘no screening’ as the current screening guidelines are voluntary for asymptomatic participants. Furthermore, we compared the cost‐effectiveness of primary tests including FIT, COLOTECT, and colonoscopy to ‘no screening’ strategy in this study. The comparators were selected based on current clinical guidelines, standard practices, and availability in the healthcare system. The US guidelines were utilized in study [34]. Our recent studies also showed supportive evidence for the implementation of the US guidelines in Hong Kong [35, 36]. The details of the comparators are listed as follows:

#### 2.2.3 Strategy 1: FIT as the Primary Screening Test

Individuals with negative results were subjected to annual repeat testing, while those with positive results were offered colonoscopy follow‐up. Subjects have conducted FIT after 10 years if normal colonoscopy results were reported as followed current Cancer Expert Working Group on Cancer Prevention and Screening (CEWG) recommendations on CRC screening from the Centre for Health Protection, The government of Hong Kong Special Administrative Region [37]. In contrast, participants with advanced colorectal neoplasm (ACN) removed would undergo surveillance colonoscopy every 3 years until no further ACN were detected. The application of the same surveillance interval for all ACN in this study was justified by the need for simplification in protocol, allowing for clearer comparisons of cost‐effectiveness among different screening strategies.

#### 2.2.4 Strategy 2: COLOTECT as the Primary Screening Test

Each subject has given one COLOTECT test after registering for this study. Among those with negative results, the test was repeated on a yearly basis, while subjects with positive results had offered colonoscopy follow‐up. The subject has conducted COLOTECT after 10 years if normal colonoscopy results were reported. The screening time interval was set due to currently no guideline for average‐risk patients in COLOTECT, and the progression of colorectal cancer development usually takes up to 10 to 15 years [38]. In contrast, participants with ACN removed would undergo surveillance colonoscopy every 3 years until no further ACN was detected. The application of the same surveillance interval for all ACN in this study was justified by the need for simplification in protocol, allowing for clearer comparisons of cost‐effectiveness among different screening strategies.

#### 2.2.5 Strategy 3: Colonoscopy as the Primary Screening Test

Each screening participant received a colonoscopy at the initial stage. Subjects who had normal colonoscopy results have been advised to have a repeat colonoscopy after a period of 10 years. On the other hand, individuals who had ACN removed during the screening process have undergone surveillance colonoscopy every 3 years until no further ACN was detected. The repeat colonoscopy was scheduled 10 years later if no additional ACN were found. The application of the same surveillance interval for all ACN in this study was justified by the need for simplification in protocol, allowing for clearer comparisons of cost‐effectiveness among different screening strategies.

#### 2.2.6 Perspective

The perspective of this health economics study is from the public health system. This choice was made to align the analysis with the broader implications for healthcare policy and resource allocation within the healthcare system. By adopting this perspective, we aim to evaluate the cost‐effectiveness of colorectal cancer screening strategies, such as COLOTECT, from the viewpoint of public health authorities who bear the costs associated with screening programs and treatment outcomes.

#### 2.2.7 Time Horizon

In this study, we chose a time horizon that extends from age 50 until age 75, which is relevant given the typical age of onset for colorectal cancer and the recommended screening guidelines. This period allows for a comprehensive assessment of the long‐term benefits and costs associated with different screening methods, ensuring that the evaluation reflects the true impact on patient outcomes and healthcare resources over time.

#### 2.2.8 Studies on Diagnostic Accuracy of COLOTECT

The sensitivity and specificity of FIT in detecting CRC were 73.0% and 91.9% [21, 39], respectively. The sensitivity and specificity of COLOTECT were set at 88.0% and 92.0% [18]. The compliance rates of FIT (60.0%), COLOTECT (96.98%), and Colonoscopy were estimated based on previous studies [21, 36], respectively. The polypectomy rate was estimated based on the progress report of the Hong Kong CRC screening pilot programme [40]. Polypectomy bleeding, perforation rates, and mortality due to perforation in colonoscopy were extracted from a meta‐analysis of post‐colonoscopy complications [41]. If a positive result was reported on COLOTECT or FIT, the compliance rate for colonoscopy was 100% (Table 1) [42]. Screening intervals were based on a guideline from the United States [43].

**TABLE 1:** Baseline estimates for the screening strategies.

	Estimate	Reference
Sensitivity of FIT (cutoff value = 20 μg/g) in detecting colorectal cancer	73.0%	[21]
Specificity of FIT (cutoff value = 20 μg/g) in detecting colorectal cancer	91.9%	[39]
Sensitivity of COLOTECT in detecting colorectal cancer	88.0%	[18, 21]
Specificity of COLOTECT in detecting colorectal cancer	92.0%	[18, 36]
Compliance rate of FIT	60.0%	[21]
Compliance rate of COLOTECT	96.98%	[21, 36]
Compliance of Colonoscopy	98.9%	[36]
Compliance rate of Colonoscopy after a positive result	100%	[42]
Rate of polypectomy of ACN (FIT & COLOTECT)	14.05%	[30, 44]
Polypectomy bleeding rate	0.98%	[41]
Polypectomy perforation rate	0.08%	[41]
Morality due to perforation	0.0029%	[41]
Cancer prevented by FIT	21.0%	[45]
Cancer prevented by Colonoscopy	54.0%	[46]
Cancer prevented by COLOTECT	25.0%	[38]
Staging of CRC at diagnosis		
I	11.2%	[30]
II	24.5%	[30]
III	31.5%	[30]
IV	32.8%	[30]
Annual mortality of CRC patients at various stages of disease (1 year)		
I	1.0%	[30]
II	4.5%	[30]
III	8.7%	[30]
IV	43.0%	[30]

Abbreviations: ACN, advanced colorectal neoplasm; CRC, colorectal cancer; FIT, faecal immunochemical tests.

#### 2.2.9 Background of COLOTECT

The COLOTECT methylation marker panel, which consists of ADHFE1, SDC2, and PPP2R5C, is utilized for non‐invasive diagnosis of colorectal neoplasia. The test kit includes a stool sample tube, enabling individuals to collect fecal materials at home, similar to the process of FIT. Once collected, the test kits are sent to designated clinics and subsequently to an accredited laboratory for analysis.

#### 2.2.10 The Treatment Options for CRC Patients in Hong Kong

The assumption of annual mortality rates of CRC diagnosed was referenced from the Hong Kong Cancer Registry [47]. Stage I and II CRC patients undergo surgery aiming for a recurrence‐free period of 3–5 years. Stage III CRC patients receive adjuvant chemotherapy after surgery, with a 70% expected cure rate. However, the remaining may experience recurrence and ultimately succumb to the disease. Stage IV patients are typically offered palliative colorectal surgery followed by palliative chemotherapy. Among these patients, approximately half of them require additional surgery to address liver metastasis [48, 49]. The model assumes that patients with stage III and IV diseases receive FOLFOX for 6 months and FOLFOX + bevacizumab for 10 months [50, 51, 52]. If the patients have considered cured of CRC, no further surveillance cost is required.

#### 2.2.11 Selection of Outcomes

The total cost per life‐years saved and Incremental cost‐effectiveness ratio (ICER) of each screening strategy were used to evaluate the cost effectiveness, using no screening strategy as the reference. In addition, total loss of cancer‐related life years and Life‐years saved were used to evaluate the benefits and harms of each strategy.

#### 2.2.12 Valuation of Outcomes

The study population comprises 100,000 average‐risk individuals aged 50 to 75, with outcomes measured using a Markov model to calculate the total cost per life‐year saved and the Incremental cost‐effectiveness ratio (ICER) based on utility values derived from existing literature relevant to the Asian population.

#### 2.2.13 Measurement and Valuation of Resources and Costs

In this study, US dollars have been used as the unit for estimating the cost. The cost component includes the direct cost of the CRC screening test, the cost of cancer treatment, the cost of investigation, and hospitalisation costs in Hong Kong (Table 2) [21, 39, 53]. In addition, diagnostic and preoperative evaluation costs include colonoscopy, histopathological examination, abdomen CT contrast scan, and outpatient specialist clinic fees. The ultrasound of the abdomen and outpatient specialist clinic were counted as types of follow‐up costs. In addition, Hospitalisation costs include one‐time device use as well as labour costs and routine inpatient care fees, while surgical fees and consultation fees, CT and PET scan fees are calculated separately. In this model, the average length of hospital stay for surgical patients is 9 days [51]. For a more accurate estimate, the indirect costs of healthcare services such as lost productivity, transportation costs, and blood transfusion costs were excluded.

**TABLE 2:** Estimates for the costs based on different screening strategies and treatment methods.

Cost item	Baseline value (US$)	Reference
One kit of FIT	19	[21]
COLOTECT	60	[31, 54]
Colonoscopy	1259	[31, 54]
Consultation fee	96	[31, 54]
Bleeding	3320	[31, 54]
Histopathological examination	142	[31, 54]
Perforation	10 790	[31, 54]
Treatment for the stage I of CRC	17 071	[31, 54]
Diagnosis	6091	[31, 54]
Treatment	10 377	[31, 54]
Follow‐up	603	[31, 54]
Treatment for the stage II of CRC	19 755	[31, 54]
Diagnosis	6091	[31, 54]
Treatment	13 061	[31, 54]
Follow‐up	603	[31, 54]
Treatment for the stage III of CRC	26 883	[31, 54]
Diagnosis	6091	[31, 54]
Treatment	20 189	[31, 54]
Follow‐up	603	[31, 54]
Treatment for the stage IV of CRC	45 115	[31, 54]
Diagnosis	38 422	[31, 54]
Treatment	13 061	[31, 54]
Follow‐up	603	[31, 54]

Abbreviations: CRC, colorectal cancer; FIT, faecal immunochemical tests.

#### 2.2.14 Currency, Price Date, and Conversion

All resource quantities and costs are based on data collected from the Hong Kong health system for the year 2022. For any costs that required conversion from Hong Kong Dollars (HKD) to USD, we utilized an exchange rate of 1 HKD = 0.13 USD as of December 31, 2022.

#### 2.2.15 Discount Rate

All future costs arising from the screening or care of CRC and all future life‐years saved through screening were discounted at an annual rate of 3% based on the guideline developed by Drummond and Jefferson (1996) [55].

#### 2.2.16 Rationale and Description of Model

The Markov models were utilized to provide a comprehensive cost‐effectiveness evaluation. The model provided flexibility, ability to incorporate probabilities, and suitability for complex health scenarios made it a preferred choice for many researchers and decision‐makers in public health and healthcare policy. The Markov model adopted in this study followed a previous CEA conducted by Dr. J. J. Y. Sung and colleagues. The sensitivity and specificity of the tests were incorporated into the model by using these parameters to determine the probabilities of true positive and true negative results for each screening strategy.

### 2.3 Analytics and Assumptions

#### 2.3.1 Cost‐Effectiveness Analysis

Effectiveness of screening is measured in terms of life‐years saved through the prevention of CRC and improved survival of earlier cancer diagnosis. Years of life lost were estimated using the Hong Kong standard life table. The life‐years lost by the age‐dependent proportions of patients dying prematurely of CRC are accumulated for each cycle during the entire expected lifetime. The number of life‐years saved because of screening corresponds to the difference in life‐years lost from cancer‐related deaths between a Markov model with and one without screening. The main outcome of this study was the incremental cost‐effectiveness ratio (ICER) between the screening strategies, that is, the cost difference divided by the difference in effectiveness between strategies. ICER is a measurement to quantify the amount of additional cost required for per life‐year saved.

#### 2.3.2 Sensitivity Analysis

As the health cost varies in different Asian countries, sensitivity analyses on ICER were conducted to assess their robustness across different intervals of key parameters, including compliance rates of screening tests, sensitivity of FIT, and cost of colonoscopy. The one‐way sensitivity analyses on the ICER were performed between different screening strategies over the possible range of model variables. The compliance rates on the initial, repeated, and follow‐up screening were assumed to be the same. The threshold values for the change of conclusion were presented if the results of sensitivity analyses are not robust. All calculations were simulated by using Excel spreadsheets.

#### 2.3.3 Characterizing Uncertainty

The uncertainty of the parameter and the Markov model was assessed by the sensitivity analysis to identify how the input parameters affect the outcomes of the model. Details have been listed in the previous section.

## 3 Result

Table 3 reported the results of no screening, FIT, COLOTECT, and Colonoscopy strategies. The 3% yearly discount was calculated for life‐years saved and cancer‐related life years lost. The case of CRC was prevented, and the cost was calculated based on the strategy, respectively. There will be 3233 cases of CRC and a total of 5598 cancer‐related life years lost among 50‐year‐old subjects if there is no screening program performed. The highest proportion of CRC prevented was observed in Colonoscopy (51.3%), followed by COLOTECT (39.3%) and FIT (4.5%). The model calculation indicates that colonoscopy screening could save more life‐years than FIT and COLOTECT, despite its higher cost.

**TABLE 3:** Result of 100,000 average‐risk individuals aged 50–75 years with no screening, FIT, COLOTECT, and colonoscopy for colorectal cancer.

Screening method	No screening	FIT	COLOTECT	Colonoscopy
Total number CRC cases	3233	3135	2010	1546
Total loss of cancer‐related life years	5598	5330	3361	2658
Cases of CRC prevented (compared with no screening)	0	98	1223	1687
Proportion of CRC case prevented (%)	0.0%	3.0%	37.8%	52.2%
Life‐years saved	0	268	2236	2940
Number of procedures				
FIT	0	243 825	0	0
COLOTECT	0	0	1 122 430	0
Colonoscopy	0	13 796	100 792	296 470
Diagnostic (without polypectomy)	0	11 858	86 631	255 274
Therapeutic (with polypectomy)	0	1938	14 161	41 196
Number of complications				
Bleeding	0	28	202	593
Perforations	0	11	81	235
Costs (USD)				
FIT	0	4 351 201	0	0
COLOTECT	0	0	52 093 673	0
Colonoscopy	0	17 328 013	104 629 105	313 321 417
Polypectomy	0	254 933	1 539 325	4 562 593
Bleeding	0	416 077	2 512 334	7 523 414
Perforations	0	110 388	666 538	1 974 052
Care of CRC				
Stage I	4 014 979	3 859 630	2 456 709	1 913 575
Stage II	14 417 641	13 851 915	8 806 519	6 868 928
Stage III	35 145 714	33 745 265	21 426 677	16 737 204
Stage IV	171 086 795	163 774 923	103 456 412	81 329 567
Total	224 665 128	237 692 344	297 587 292	434 230 750
Total costs per life‐years saved	—	887 299	133 079	147 716
Additional cost	—	13 027 216	72 922 164	209 565 621
ICER versus no screening	—	48 630	32 610	71 290
ICER versus FIT	—	—	30 430	73 562
ICER versus COLOTECT	—	—	—	194 245

Abbreviations: CRC, colorectal cancer; FIT, faecal immunochemical tests; ICER, Incremental cost‐effectiveness ratio.

**FIGURE 1:**
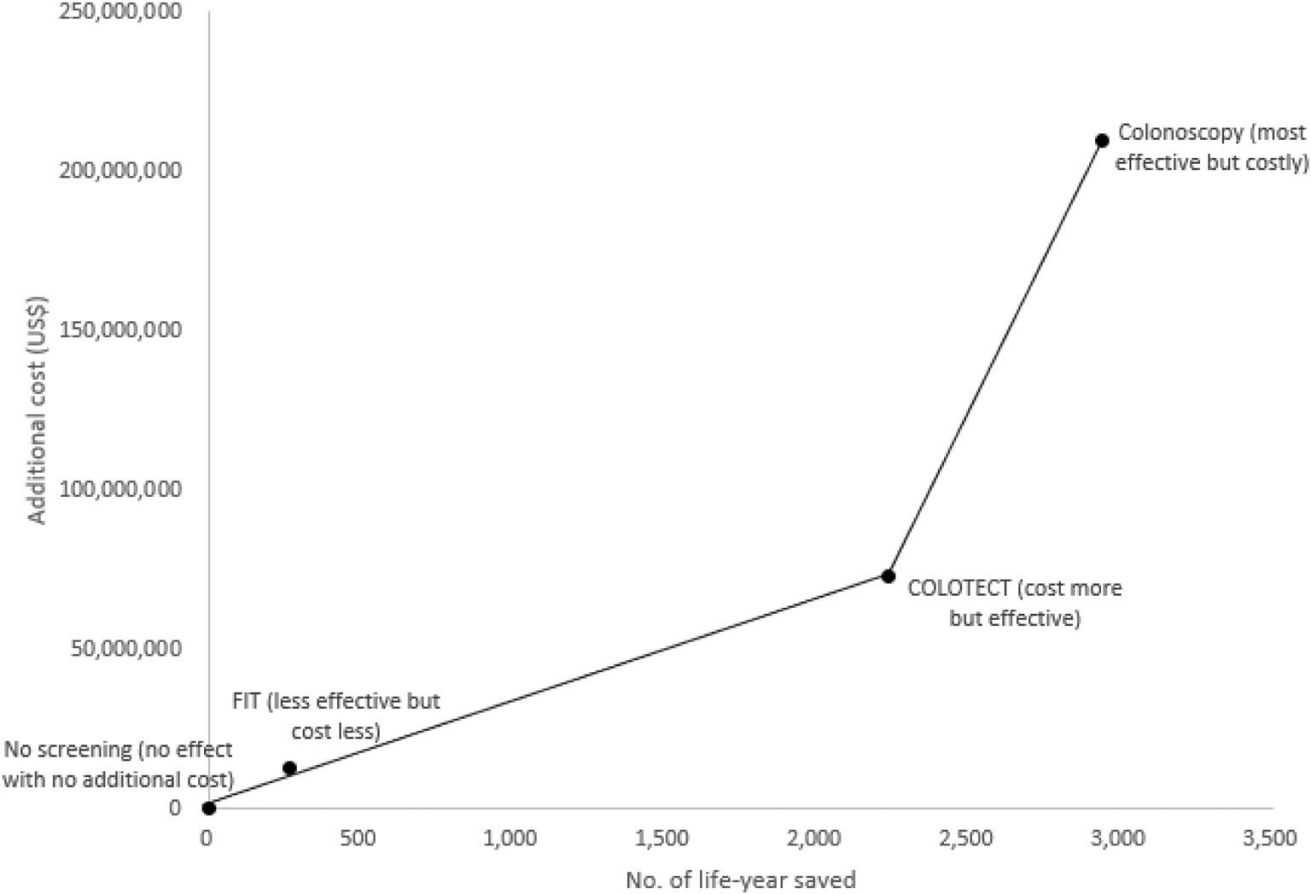
Cost‐effectiveness analysis for the screening tests. FIT: faecal immunochemical test.

**FIGURE 2:**
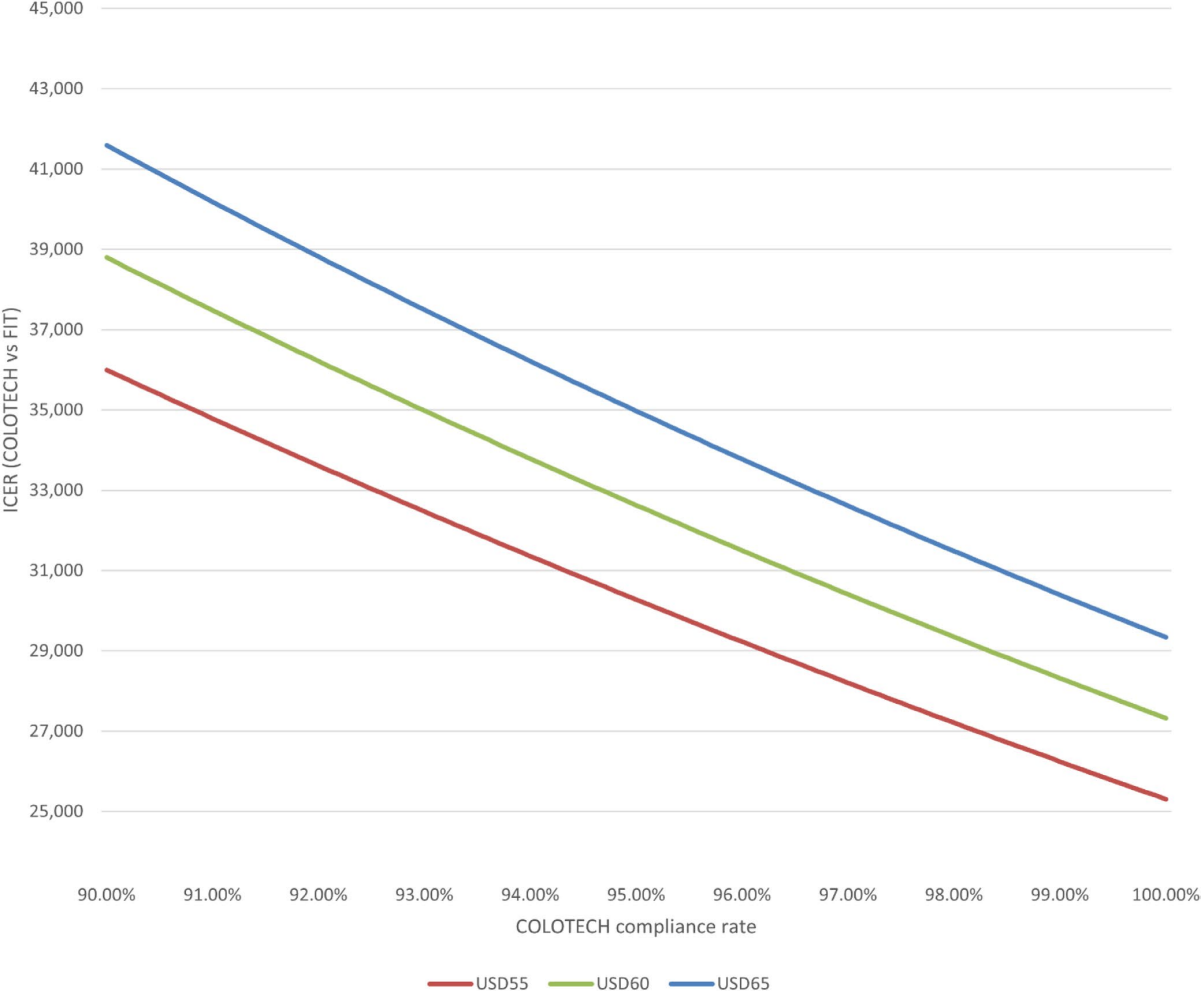
Analysis of sensitive of COLOTECT and compliance rate. FIT, faecal immunochemical test; ICER, Incremental cost‐effectiveness ratio.

The highest total cost for CRC was observed in colonoscopy (USD 434 million), followed by COLOTECT (USD 297 million), FIT (USD 238 million), and no screening (USD 225 million). However, for the cost per life‐year saved, FIT, COLOTECT, and Colonoscopy cost about USD 887,299, USD 133,079, and USD 147,716, respectively. The cost advantage of COLOTECT can be observed. When compared with no screening, the ICER of FIT, COLOTECT, and colonoscopy is USD 48,630, USD 32,610, and USD 71,290 (Table 3, Figure 1). The lowest additional cost per life‐year saved was observed in the COLOTECT strategy among the three strategies. In addition to cost considerations, cost‐effectiveness is also determined by performance. We tested COLOTECT's compliance rate and changes in ICER based on costs of USD 55, USD 60, and USD 65 respectively (Figure 2) and found that COLOTECT's ICER decreased as the compliance rate increased; this indicates the additional cost required to save a life‐year in lower compliance rates.

### 3.1 ICER Plan

**FIGURE 3:**
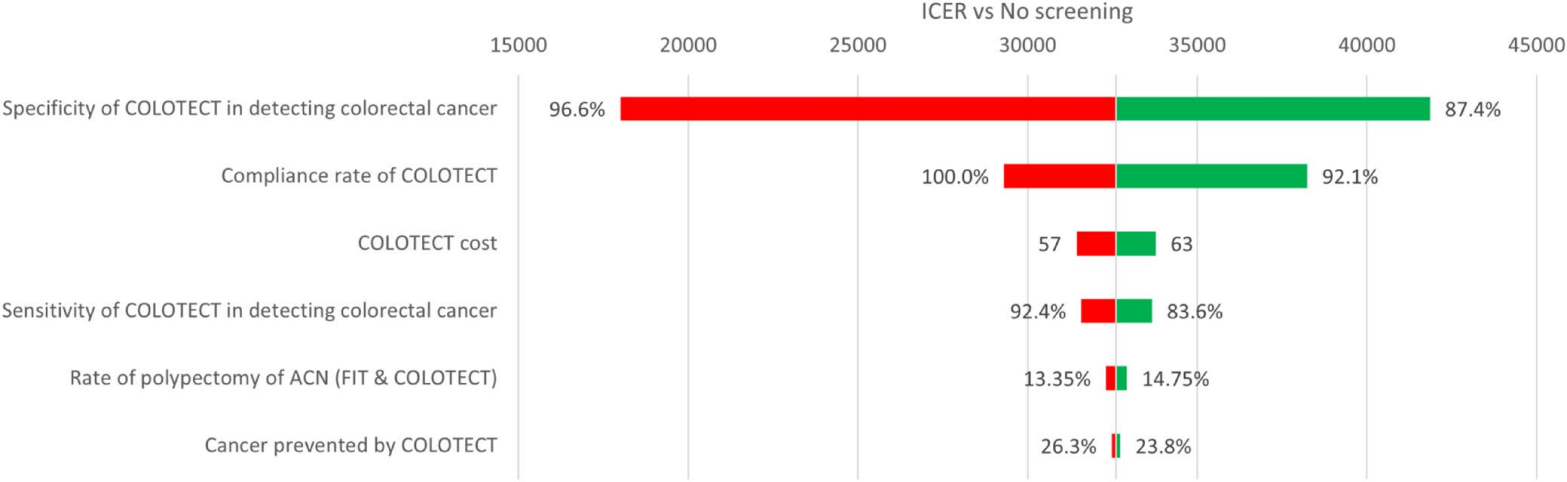
Tornado diagram of ICER difference between COLOTECT and no screening. ACN, advanced colorectal neoplasm; FIT, faecal immunochemical test; ICER, Incremental cost‐effectiveness ratio.

The plot illustrates the trade‐off between cost and effectiveness for different screening methods (Figure 3). No Screening serves as the baseline, with no additional cost and no life‐years saved. FIT (Faecal Immunochemical Test) is a cost‐effective option with moderate life‐years saved and relatively low additional costs. COLOTECT is more effective than FIT but incurs higher costs. Colonoscopy is the most effective screening method in terms of life‐years saved, but it is also the most expensive option.

### 3.2 Tornado Diagram for Sensitivity Analysis

**FIGURE 4:**
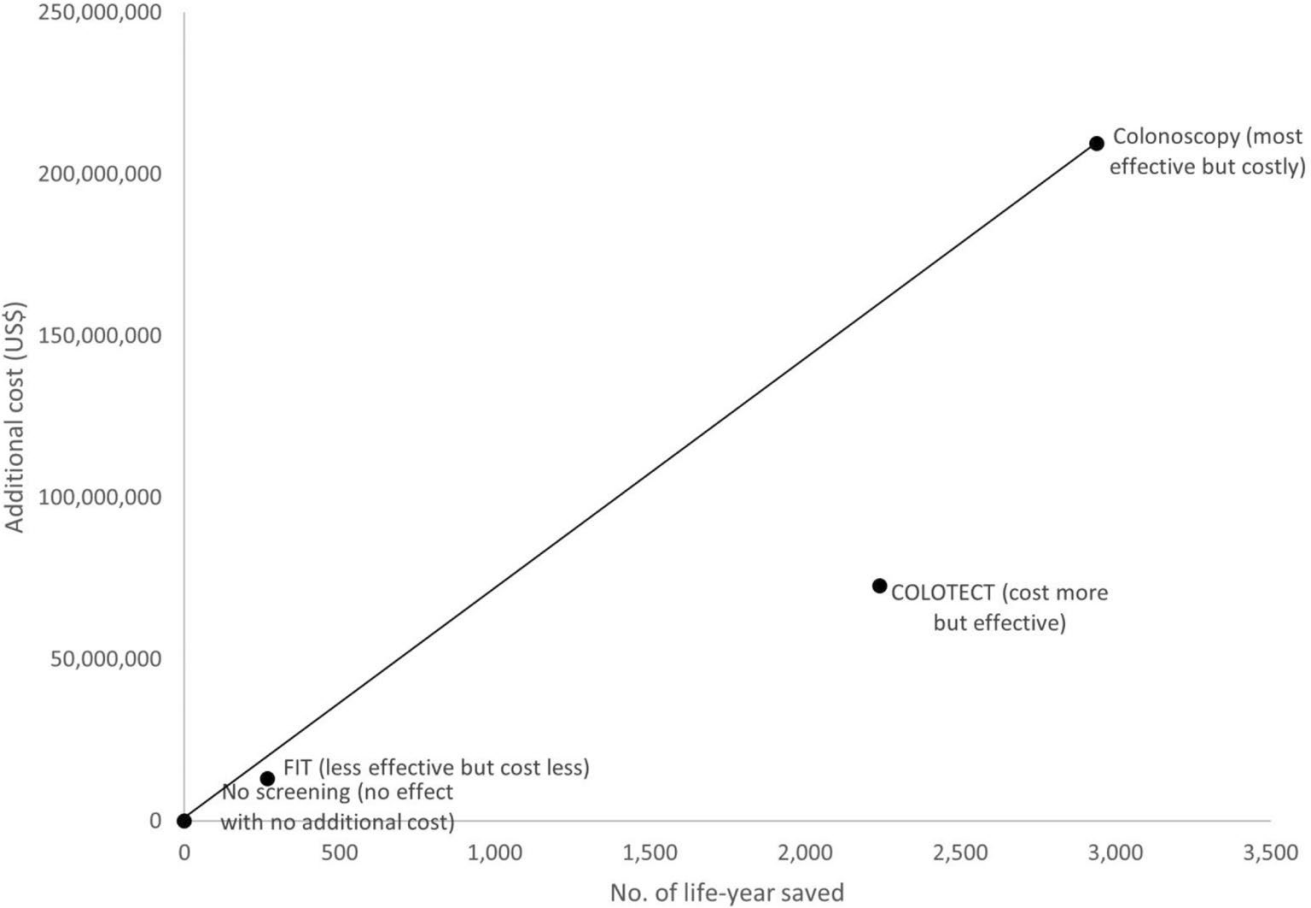
The ICER plan for this study. FIT, faecal immunochemical test.

The plot demonstrates which factors have the greatest impact on the cost‐effectiveness of COLOTECT (Figure 4), helping identify areas for optimization in screening strategies. The specificity of COLOTECT has a significant influence on ICER, with lower specificity leading to higher ICER values. Higher compliance lowers ICER, making COLOTECT more cost‐effective. The polypectomy rate and sensitivity of COLOTECT moderately influence cost‐effectiveness. The cost of COLOTECT and cancer prevention rate have a relatively smaller impact but still affect ICER.

### 3.3 Effect of Uncertainty

The cost associated with CRC screening or care, as well as all future life‐years saved through screening, was discounted at an annual rate of 3% based on the guideline developed by Drummond and Jefferson (1996) [55]. The age‐specific incidence of CRC for the general population without screening was retrieved from the Hong Kong Cancer Registry for the quinquennial age range from 50 to 75 years old among 2010–2019 [56]. The mortality rate for each age is based on the previous CEA studies conducted by Tsoi and colleagues [31].

## 4 Discussion

### 4.1 Major Findings

This study compares FIT, COLOTECT, and colonoscopy screening methods in a cost‐effective dimension and provides information for better policy making for CRC screening. First, this COLOTECT (ICER: USD 32610) as the main screening method is more cost‐effective relative to colonoscopy (ICER: USD 71290) and FIT (ICER: USD 48630) due to the lowest ICER. Second, in contrast, COLOTECT's compliance rate (96.98%) was higher than FIT (60%), and COLOTECT's sensitivity (88.0%) and specificity (92.0%) were also higher than FIT (Sensitivity:73%; Specificity: 91.9%), signifying that COLOTECT has higher performance and effectiveness than FIT. In addition, the total cost of per life year saved for COLOTECT is lowest among the three strategies. As a result, our study points to COLOTECT as the most cost‐effective among FIT, COLOTECT, and Colonoscopy, saving the most lives at the least additional cost and preventing CRC cases at the lowest cost.

### 4.2 Cost Effectiveness Threshold

The ICERs were compared with the willingness to pay (WTP) threshold for a QALY according to the WHO guideline that interventions with a cost per QALY gained of less than 1 × GDP per capita (HK$395429 in year 2017; at US$1 = HK$7.8 = US$50696) would be considered highly cost‐effective.

### 4.3 Limitation of FIT

Our study found that FIT (ICER: 48630) may be more cost‐effective than colonoscopy (ICER: 71290), and that findings are similar to a previous systematic review.

A systematic review indicated screening with FIT every year or every 2 years can be more cost‐effective (ICER ≤ $25000) than colonoscopy every 10 years, and the study found that FIT (41.6%) had a higher participation rate than colonoscopy (21.9%) [57], This may be related to the public fear of and resistance to invasive screening [6]. Another study based on a screening model for ages 50 to 75, which compared FIT with colonoscopy, computed tomography colon imaging, mtSDNA, PillCam, and mSEPT9, found that FIT was the most cost‐effective across multiple screening strategies. Moreover, FIT resulted in a 26% cost savings and a 63% reduction in screening burden compared with colonoscopy [58].

Although FIT has been proven to be a more cost‐effective strategy among different screening methods in the previous study [58], the limitations of FIT for CRC screening cannot be ignored. A review study indicated that FIT was 71%–75% sensitive for CRC detection and 27%–29% sensitive for precancerous lesions only [59]. Another research study also found that FIT cannot detect non‐advanced adenomas [60]. Furthermore, our study also found that FIT had the lowest compliance rate of the three screening strategies, at 60%, compared with 96.98% for COLOTECT and 98.9% for colonoscopy. ICER may change due to the impact of compliance rate, so a stable or low sensitivity to compliance rate can help avoid possible cost losses in large‐scale screening. In addition, the specificity of FIT is not as good as that of other screening strategies, which may lead to false‐positive results [61]. In a Canadian population‐based study, the predictive ability of FIT for colorectal adenomas was insufficient (AUC: 0.60, 95% CI: 0.54–0.65), and the sensitivity and specificity of FIT for advanced adenomas were 49.5% and 62.7% at a critical level of cutoff of 75 ng/mL [62].

### 4.4 Strength of COLOTECT

COLOTECT, a multi‐target fecal DNA‐based non‐invasive test, had a higher detection rate than FIT in detecting CRC and advanced adenomas. The COLOTECT detects CRC through SDC2, ADHFE1, and PPP2R5C. The performance of multi‐biomarker DNA testing has long been validated, and several fecal DNA‐based markers such as SFRP2, NDRG4, SDC2, and TFPI2 have been recognized as potential biomarkers for CRC detection [63, 64, 65, 66]. Another study also verifies our result of performance for COLOTECT. A clinical trial conducted in China showed single DNA biomarkers of SDC2, ADHFE1, and PPP2R5C had sensitivities of 81.9%, 81.9%, and 71.4%, and specificities of 99.0%, 90.0%, and 95.0% for CRC, respectively, and the sensitivities for precancerous lesions were 40.0%, 43.9%, and 54.0%, respectively. More importantly, the study tested the combination of SDC2, ADHFE1, and PPP2R5C, which showed an AUC = 0.930 (95% CI: 0.889–0.970) for CRC with a sensitivity of 84.8% and a specificity of 98.0%, and AUC = 0.632 (95% CI 0.542–0.723) for precancerous lesions, with a sensitivity of 31.6% and a specificity of 92.0%, and the study indicated that the combined score of these three microbial markers was more sensitive than FIT in the diagnosis of CRC and advanced adenoma [46]. This study demonstrates the performance of COLOTECT as it uses the same genes for combined testing and also validates that multi‐biomarker combined testing outperforms single‐gene testing. Other studies have also verified that these three microorganisms perform better than FIT in verifying CRC [67, 68]. These studies demonstrate that COLOTECT has better validation and performance than FIT in diagnosing rectal adenomas and CRC. While some studies point to compliance issues with stool‐based biomarker testing [69, 70]. However, the COLOTECT achieved a compliance rate of 96.98%, much higher than FIT's 60%. Further studies should be conducted to examine the compliance with COLOTECT and multi‐target DNA screening tests in other populations.

This research points out that the COLOTECT, as a primary screening method for large‐scale CRC screening, is more cost‐effective than FIT and also colonoscopy. The ICER of COLOTECT is 25% lower than FIT, which means that each life‐year can be saved at a lower additional cost, and also the performance of COLOTECT is higher than that of FIT. Additionally, the ICER for colonoscopy (USD160,808) is 2 times that of COLOTECT, and the increased costs may be caused by the invasive nature, lower acceptance, and unstable quality [71]. Recent literature has presented the possibility of using fecal biomarkers as alternative screening tests to replace FIT due to the better performance of diagnostic sensitivity [68], and the higher acceptability of stool‐based testing CRC screening also further supports this perspective [72]. Therefore, we have reason to believe that COLOTECT is more cost‐effective and highly acceptable as the main primary screening method. Further follow‐up qualitative and quantitative study could be conducted to assess the acceptance and perception of the population towards the COLOTECT screening method.

### 4.5 Limitation

The study has several limitations that should be acknowledged. First, there is no consideration of potential heterogeneity in outcomes across subgroups, such as age, gender, and socioeconomic status, which may impact the generalizability of the findings. Furthermore, there was no engagement with patients, service recipients, or stakeholders during the study design, which may have resulted in a lack of relevance to the needs and perspectives of those most affected by colorectal cancer.

Although the assumption used in the study was based on the evidence from the previous study, the study did not include the diagnostic accuracy of the different screening strategies in its calculations, and the clinical evidence we used to test the screening strategies, especially the rate of adherence, was not yet reflected in our study. Future studies are necessary to consider compliance rates as well as diagnostic accuracy when examining cost‐effectiveness.

In addition, the application of the same surveillance interval for all polyps in this study was justified by the need for simplification in protocol, allowing for clearer comparisons of cost‐effectiveness among different screening strategies; however, we admitted that this approach also represents a limitation, as it may overlook the variability in progression risk associated with different types of polyps, which could affect individual patient outcomes. Moreover, most of the CEAs used quality‐adjusted lifeyears (QALY) as the common threshold. However, the QALY was not included in this study due to a lack of data on Quality of Life. This study also did not include consideration of subgroups and the distributional effects due to the mixing of data sourced from multiple databases and studies incorporated in the analysis.

The quality of life, as defined by WHO, is An individual's perception of their position in life in the context of the culture and value systems in which they live and in relation to their goals, expectations, standards, and concerns [73]. The quality of life was not included in this study as in order to access the above‐mentioned information, we may need to conduct a comprehensive qualitative and/or quantitativetudy.

The study acknowledges the limitation of not engaging patients, service recipients, or stakeholders in its design. There were no formal approaches to involve the general public, communities, or relevant stakeholders, which may impact the relevance and applicability of the findings. Future research should prioritize patient and community engagement to ensure that the study design reflects the needs and perspectives of those affected by colorectal cancer.

## 5 Conclusion

The study compared the cost‐effectiveness of FIT, COLOTECT, and Colonoscopy. COLOTECT, using a multi‐target biological combination test, was found to be the most cost‐effective screening strategy, significantly reducing cancer‐related life‐years lost, preventing an increase in the number of CRC cases, and saving year‐life at a lower additional cost relative to other strategies.

## Author Contributions

**Junjie Huang:** conceptualization (equal), supervision (equal), writing – original draft (equal). **Mingtao Chen:** writing – original draft (equal). **Victor C. W. Chan:** data curation (equal), formal analysis (equal). **Xianjing Liu:** writing – review and editing (equal). **Chaoying Zhong:** writing – review and editing (equal). **Jianli Lin:** writing – review and editing (equal). **Junjie Hang:** writing – review and editing (equal). **Claire Chenwen Zhong:** writing – review and editing (equal). **Jinqiu Yuan:** writing – review and editing (equal). **Martin C. S. Wong:** conceptualization (equal), supervision (equal), writing – review and editing (equal).

## Ethics Statement

Survey and Behavioural Research Ethics (No. SBRE‐22‐0454), The Chinese University of Hong Kong, Hong Kong SAR.

## Consent

The authors have nothing to report.

## Conflicts of Interest

The authors declare no conflicts of interest.

## Supporting information


**Figure S1.** Markov process on strategies using FIT, COLOTECT and colonoscopy as primary screening test.

## Data Availability

The datasets used and/or analysed during the current study are available from the corresponding author on reasonable request.
