# Supplementary material for: The Cost‐Effectiveness of a Multi‐Target Stool DNA‐Based Screening (COLOTECT), FIT, Colonoscopy and No Screening for Colorectal Cancer
Source: Cancer Rep (Hoboken). 2025 Apr 24;8(4):e70176. doi: 10.1002/cnr2.70176 (PMC12021674; doi:10.1002/cnr2.70176)
Supplement: Supplementary file 1 — Figure S1. Markov process on strategies using FIT, COLOTECT and colonoscopy as primary screening test. [file CNR2-8-e70176-s001.docx]

**Supplementary Figure 1.** Markov process on strategies using FIT, ColoTect and colonoscopy as primary screening test

1. FIT / ColoTect


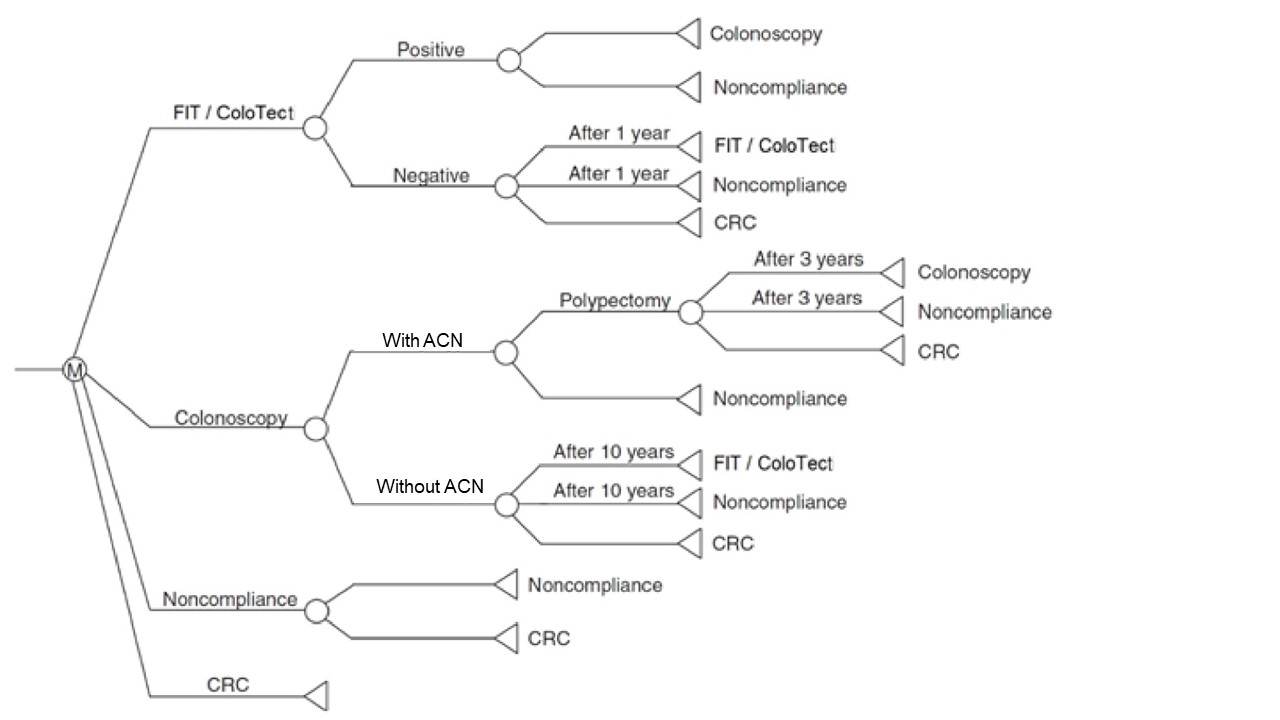


1. Colonoscopy


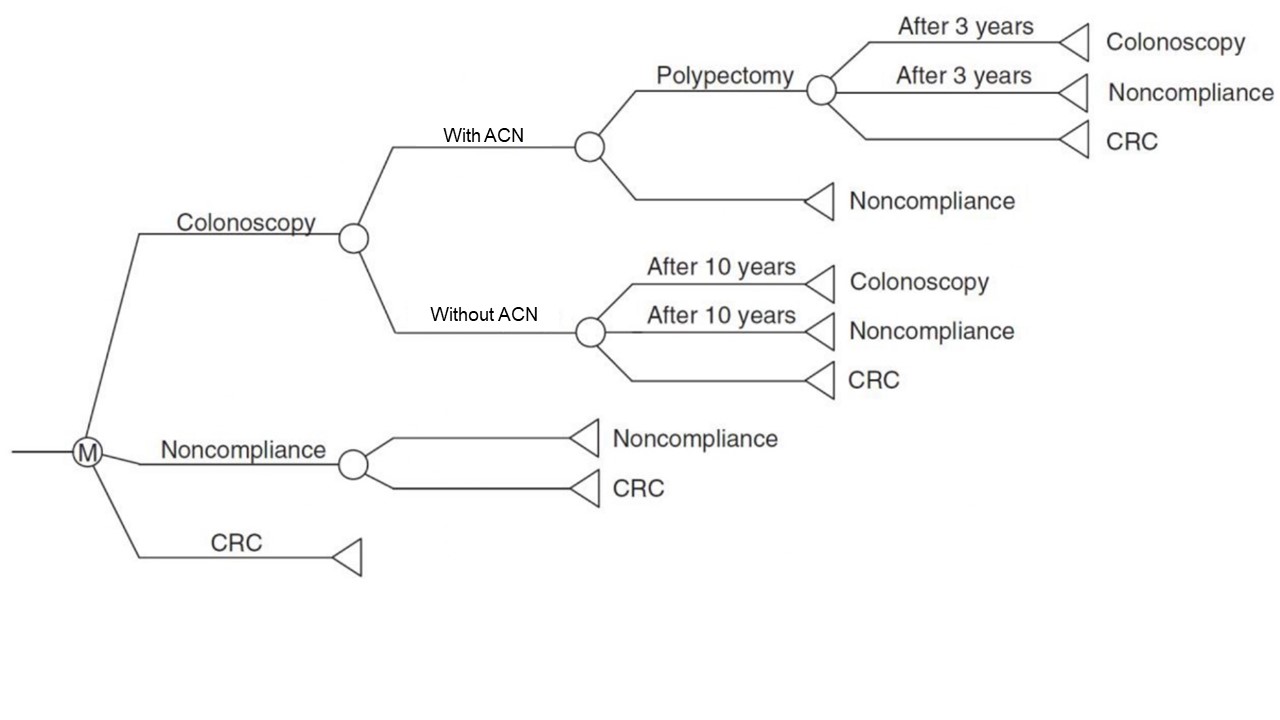


ACN: advanced colorectal neoplasm; CRC: colorectal cancer; FIT: faecal immunochemical tests;
